# High-density genetic map construction using whole-genome resequencing of the *Cymbidium eburneum*(‘Duzhan Chun’) × *Cymbidium insigne* (‘Meihua Lan’) F1 population and localization of flower color genes

**DOI:** 10.3389/fpls.2025.1685531

**Published:** 2025-11-19

**Authors:** Yu Han, Yutong Cui, Yu Chen, Dandan Rao, Erhuan Wu, Rongcun Gan, Tengmin Li, Mi Tian

**Affiliations:** 1 Hainan Academy of Forestry (Hainan Academy of Mangrove) Forestry High-tech Research Institute, Haikou, China; 2 The Innovation Platform for Academicia, Haikou, Hainan, China

**Keywords:** flower color, whole-genome resequencing, genetic linkage map, SNP, genome-wide association study

## Abstract

As a traditional and valuable ornamental flower in China, Cymbidium orchids exhibit significant developmental potential in the floriculture industry due to their profound cultural connotations and unique aesthetic characteristics. Flower color diversity, as one of the most important ornamental traits, not only attracts extensive attention in the breeding and development of new varieties but also plays a critical role in the evolution of floral traits and ecological functions. Color changes during the flowering process of Cymbidium are common; however, the genetic regulatory networks underlying these dynamics remain insufficiently understood. In this study, an F1 hybrid population consisting of 150 individuals derived from a cross between *Cymbidium goeringii (‘Duzhan Chun’)* and *Cymbidium insigne (‘Meihua Lan’)* was employed. Using whole-genome resequencing at an average depth of 5×, a high-density genetic linkage map was constructed. The sequencing data exhibited excellent quality (Q30 ≥ 96%), and after stringent quality control, 2,306,434 high-quality SNPs were retained. Ultimately, a genetic map comprising 7,734 bin markers was established, spanning a total genetic distance of 255.945 cM with an average marker interval of 1.19 cM. Genome-wide association study (GWAS) identified 121 SNP loci significantly associated with flower color (P < 1 × 10⁻^5^), which were predominantly enriched in carotenoid biosynthesis and phenylpropanoid metabolic pathways. Candidate gene analysis revealed that 9-cis-epoxycarotenoid dioxygenase (NCED), the MYB60 transcription factor, carotene epsilon-monooxygenase (LUT1), and the WRKY6 transcription factor likely influence flower color formation by regulating pigment synthesis and accumulation. This study not only establishes the highest-density genetic linkage map for Cymbidium to date but also systematically elucidates the genetic basis of flower color variation, providing critical theoretical foundations and molecular marker resources for molecular breeding in Cymbidium.

## 1 Introduction

Flower color is one of the most important phenotypic traits in ornamental plants, as it not only determines their aesthetic value but also plays a crucial role in ecological adaptation and evolution. The diversity of flower colors results from the combined effects of natural selection, genetic mutations, and ecological interactions. Pollinator preference constitutes a major selective pressure driving flower color differentiation; for example, bees are typically attracted to blue and purple flowers, whereas birds prefer red and orange flowers (Rausher, 2008). The formation of flower color involves the biosynthesis, transport, and deposition of pigments such as flavonoids, carotenoids, and betalains, in addition to regulation by environmental factors including light and temperature (Tanaka and Brugliera, 2013; Lee et al., 2023). At the molecular level, the flavonoid biosynthetic pathway represents the primary metabolic route, producing anthocyanins, flavonols, and flavones that accumulate in petal cells to generate diverse colors (Grotewold, 2006). Mutations or regulatory changes in key genes frequently underlie phenotypic variation, such as deletion of *AN2* in petunia leading to white flowers (Quattrocchio et al., 1999) or high expression of *FLS* in chrysanthemum reducing anthocyanin accumulation (Yang et al., 2023).

In recent years, advances in genomics and molecular biology have greatly expanded our understanding of flower color regulation. Integrated analyses combining whole-genome sequencing (WGS), transcriptomics, and metabolomics have become essential tools for dissecting pigment biosynthesis and regulatory networks. For example, copy number variation in *ANS* and *UFGT* was associated with petal color intensity in Osmanthus fragrans (Chen et al., 2021), and dynamic expression of *DFR* and *MYB* genes was linked to flower color transitions in Syringa oblata (Chen et al., 2022). Genome-wide association studies (GWAS), coupled with CRISPR-Cas9, have further facilitated functional validation of causal variants, such as a mutation in F3’H that regulates blue pigmentation in chrysanthemum (Costanzo et al., 2025). These advances not only deepen the understanding of pigment regulatory networks but also provide molecular targets for precision breeding.

Despite such progress, research on flower color in orchids remains limited. Most studies have focused on individual species or candidate genes, such as *MYB1* in Phalaenopsis promoting purple pigmentation through activation of *ANS* and *DFR* (Yan et al., 2021), or *FLS* in Oncidium competing for flavonoid precursors to produce white flowers (Lei et al., 2023). However, systematic genome-wide analyses of flower color variation in orchid hybrid populations are lacking. Moreover, large genome sizes (1–5 Gb), high heterozygosity (>1.5%), and the scarcity of chromosome-level reference genomes have hindered the construction of high-density linkage maps in orchids. To date, only medium-density maps generated by reduced-representation sequencing methods have been reported (Zhou et al., 2014), limiting the resolution required to dissect complex ornamental traits such as flower color.

**Figure 1:**
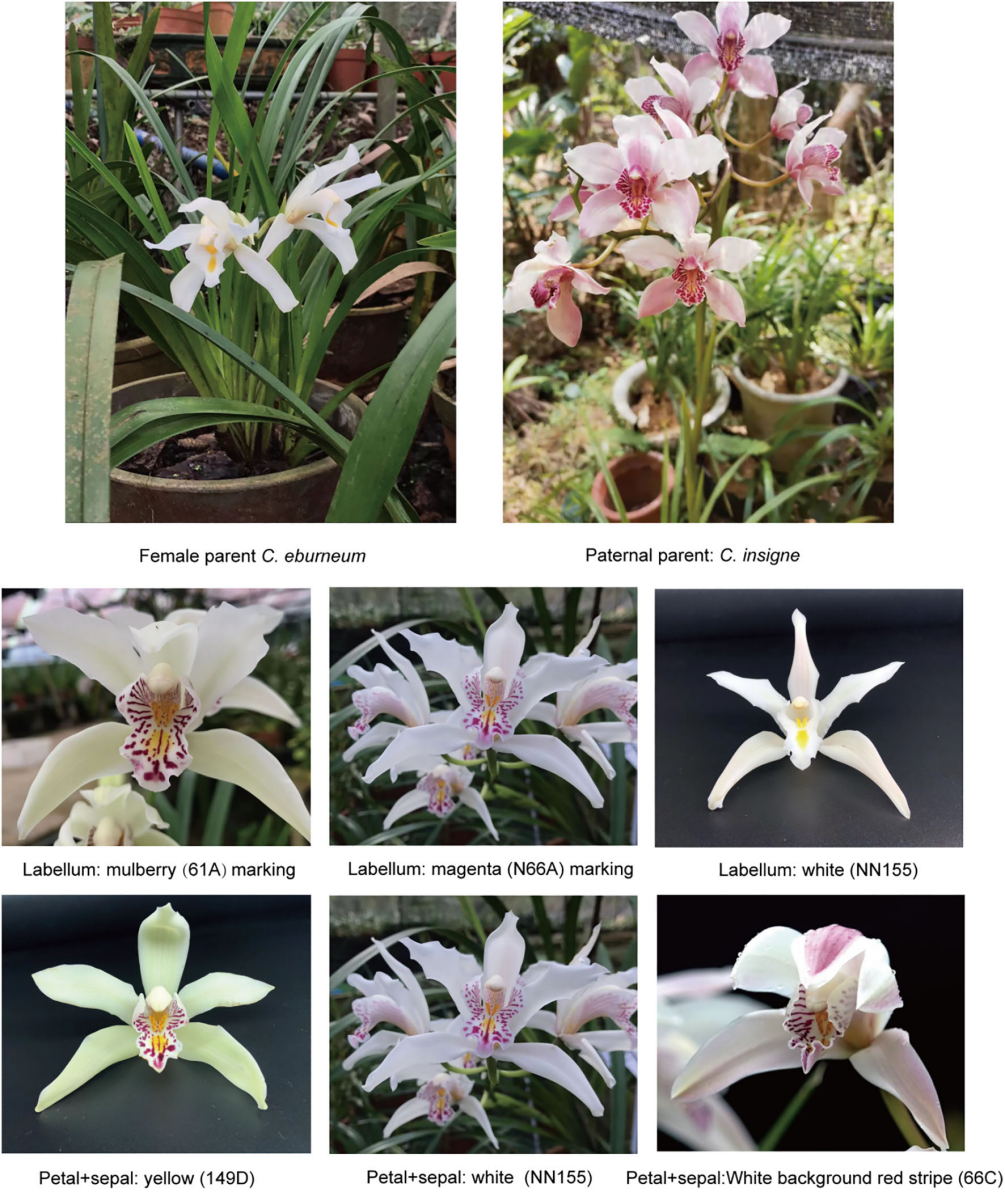
Flower color pictures of parents and offspring.

In this study, we used an F1 population derived from *C.eburneum× C. insigne*, which displays extensive variation in flower color (**Figure 1**), to investigate the genetic basis of color formation. Using whole-genome resequencing, we developed a high-density SNP-based genetic map and combined it with flower color phenotypic data to perform GWAS with a mixed linear model (MLM). Candidate genes associated with significant loci were further identified through functional annotation and validated by expression analysis. Our work provides new insights into the molecular mechanisms of flower color formation in orchids and establishes a theoretical foundation for marker-assisted breeding and targeted flower color improvement, while also demonstrating the broader potential of high-density genetic mapping in orchid genomics research.

## 2 Materials and methods

### 2.1 Experimental materials and hybrid system establishment

In 2015, wild *Cymbidium eburneum* (‘Duzhan Chun’) and *Cymbidium insigne* (‘Meihua Lan’) were crossed. Hybrid seeds were obtained in March 2017 and subsequently sown to generate an F1 population. These plants are currently maintained at the Orchid Germplasm Resource Nursery of the Yunlong Base, Hainan Academy of Forestry Sciences (19°52′21″N, 110°28′59″E). Between December 2023 and March 2024, healthy, pest- and disease-free individual F1 plants were selected during their full flowering period for sample collection. Flower surfaces were first wiped with absolute ethanol. Using a scalpel on a precooled tray, red and yellow tissues from the labellum (lip petal) were carefully dissected, along with white, yellow, and red tissues from the petals and sepals. Since the pigmented tissue layers are thin, the tissues were gently sliced with the scalpel tip to avoid contamination by non-target colors. Select 5 flowers of each color, mix the samples, and place them into 3ml pre-cooled EP tubes. Rapidly immerse in liquid nitrogen for 20 minutes, then store at -80°C. Each sample consists of 3 biological replicates for RNA-seq sequencing analysis. In December 2023, healthy and disease-free mature leaves were cut from 150 F1 generation plants, wiped with distilled water, put in liquid nitrogen for 30 seconds, and then stored in a refrigerator at -80°C.

### 2.2 DNA extraction and sequencing

Genomic DNA was extracted from the two parental lines and 150 F1 samples using the CTAB method. DNA integrity was assessed by 1% agarose gel electrophoresis, and purity was measured using a NanoDrop 2000 spectrophotometer (Thermo Fisher Scientific, USA). DNA concentration was quantified with a Qubit Fluorometer (Life Technologies, CA, USA). After obtaining high-quality genomic DNA, sequencing libraries were constructed. Whole-genome resequencing was performed on the Illumina NovaSeq 6000 platform, with sequencing depths of 10× for the two parents and 5× for the F1 samples.

### 2.3 Detection of genome-wide genetic variations

Sequencing data from 150 F1 orchid individuals were aligned to the *Cymbidium ensifolium* reference genome(https://ngdc.cncb.ac.cn/gwh/Assembly/20686/show). Low-quality bases and adapter sequences in paired-end reads were removed using Trimmomatic software (v0.39) (Bolger et al., 2014). Subsequently, the cleaned, contamination-free genomic data for each sample were mapped to the reference genome using Burrows-Wheeler Aligner MEM (BWA-MEM) (v0.7.13-r1126) with default parameters. BAM files were then sorted and PCR duplicates were marked and removed using Picard Tools (http://broadinstitute.github.io/picard) with the SortSam and MarkDuplicates functions. For SNP detection, all variants were called and filtered using the widely adopted Genome Analysis Toolkit (GATK).

### 2.4 Construction of the genetic linkage map

Following the acquisition of SNP markers, stringent filtering was applied to ensure the quality of the genetic map: (1) only markers heterozygous in at least one parent were retained; (2) low-quality markers were excluded. Based on the physical position information of each marker, all polymorphic markers were grouped into 8 linkage groups according to their respective chromosomes. The genetic linkage map was constructed using JoinMap 3.0 software.

### 2.5 Genome-wide association analysis and candidate gene identification with functional enrichment

Prior to performing genome-wide association studies (GWAS), missing genotypes in SNPs, InDels, and structural variants (SVs) were imputed using Beagle software (v5.4) (Browning et al., 2018) to ensure complete genotype datasets. To reduce false positives, GWAS for flower color-related traits in Cymbidium was conducted using the BLINK model implemented in GAPIT3 software, based on the combined set of SNPs, InDels, and SVs. Genome-wide and chromosome-wide significance thresholds were determined using the Bonferroni correction method, set as 0.05 divided by the total number of variants and 1 divided by the total number of variants, respectively. The variants considered included quality-controlled SNPs, InDels, and SVs. Manhattan and Q-Q plots were generated using the CMplot R package (v4.20). Significant SNPs, InDels, and SVs identified by GWAS were used to screen candidate genes based on the Cymbidium reference genome annotation. Using BedTools software (Quinlan and Hall, 2010), genes located within a 0.5 Mb upstream and downstream window of significant variant loci were selected as candidate genes. Subsequently, functional enrichment analysis of candidate genes was performed using the g:Profiler web tool (https://biit.cs.ut.ee/gprofiler/gost), leveraging Gene Ontology (GO) and Kyoto Encyclopedia of Genes and Genomes (KEGG) databases to elucidate the biological functions and pathways involved.

### 2.6 RNA extraction and sequencing

Total RNA was extracted from each sample using the TRIzol reagent. Equal amounts of RNA from the samples were pooled and assessed for quality using a spectrophotometer to ensure they met the library construction requirements. Following quality confirmation, mRNA was enriched and isolated using magnetic beads conjugated with Oligo (dT). The purified mRNA was then fragmented into short fragments ranging from 100 to 400 base pairs. Using these mRNA fragments as templates, first-strand cDNA was synthesized with a reverse transcription kit and random primers, followed by synthesis and purification of double-stranded cDNA. The purified cDNA underwent end repair and adenylation of 3’ ends. Size selection of fragments was performed using the AMPure XP beads kit. Subsequently, the DNA fragments were PCR-amplified and purified to construct the cDNA library. Transcriptome sequencing was carried out on the Illumina HiSeq™ 4000 high-throughput sequencing platform.

### 2.7 RNA-seq data analysis

Raw sequencing reads were subjected to quality control to remove low-quality sequences, resulting in clean reads. In this study, Trinity software was used to assemble the clean reads by leveraging sequence overlap information to generate contigs, which were subsequently assembled into transcripts. Clean reads were then rapidly and accurately aligned to the Cymbidium reference genome using the STAR (Spliced Transcripts Alignment to a Reference) software. Gene expression levels in each sample were quantified using HTSeq (v0.5.4 p3), with fragments per kilobase of transcript per million mapped reads (FPKM) values representing gene expression abundance across different samples. Differentially expressed genes (DEGs) were identified using the criteria of false discovery rate (FDR) < 0.05 and |log_2_ fold change| > 1. Functional annotation of DEGs was performed via Gene Ontology (GO) and Kyoto Encyclopedia of Genes and Genomes (KEGG) analyses, enabling the identification of genes associated with flower color in orchids.

### 2.8 qPCR validation of flower color-related genes

Five Cymbidium F_1_ samples representing different flower colors were selected for RNA extraction using a plant RNA extraction kit (RC113-01, Nanjing Novizan Biotechnology Co., Ltd.). RNA purity and concentration were assessed with a NanoDrop 2000 microvolume spectrophotometer (Thermo Fisher Scientific, USA). Reverse transcription of RNA was performed using the Ac⁃cuRT Genomic DNA Removal Reverse Transcription Kit (Applied Biological Materials Inc., Canada). The synthesized cDNA served as the template for RT-qPCR assays. Act3 was selected as the internal reference gene, and seven key candidate genes associated with flower color were analyzed. To ensure reliability, each assay included three biological replicates and three technical replicates. Primers were designed based on the Oligo7 basic algorithm; details are provided in **Table 1**. The RT-qPCR reaction volume was 10 μL, comprising 0.5 μL of template, 0.25 μL each of forward and reverse primers, 5 μL of SYBR Green qPCR Master Mix, with the remainder made up with nuclease-free water. Relative expression levels of target genes were calculated using the 2^^−ΔΔCt^ method. Statistical analyses and graphical representations were performed using SPSS 25.0 and GraphPad Prism 5.0 software.

**Table 1:** Statistics on the basic information of the map.

Linkage group ID	Total marker	Distance(cM)	Average distance(cM)
LG1	45	72.845	1.618777778
LG2	16	14.882	0.930125
LG3	57	46.99	0.824385965
LG4	23	51.257	2.228565217
LG5	34	25.347	0.7455
LG6	13	11.492	0.884
LG7	17	20.962	1.233058824
LG8	9	12.17	1.352222222
Total	214	255.945	1.196004673

Linkage group ID: The identifier of the linkage group, corresponding one-to-one with the genome numbering in this project; Total Marker: The number of markers shown above, representing the total number of markers on a linkage group; Total distance: The total genetic distance, representing the sum of the genetic distances of markers on a linkage group; Average distance: The average genetic distance, representing the mean genetic distance between markers on a linkage group.

## 3 Results

### 3.1 Whole-genome resequencing data analysis

**Figure 2:**
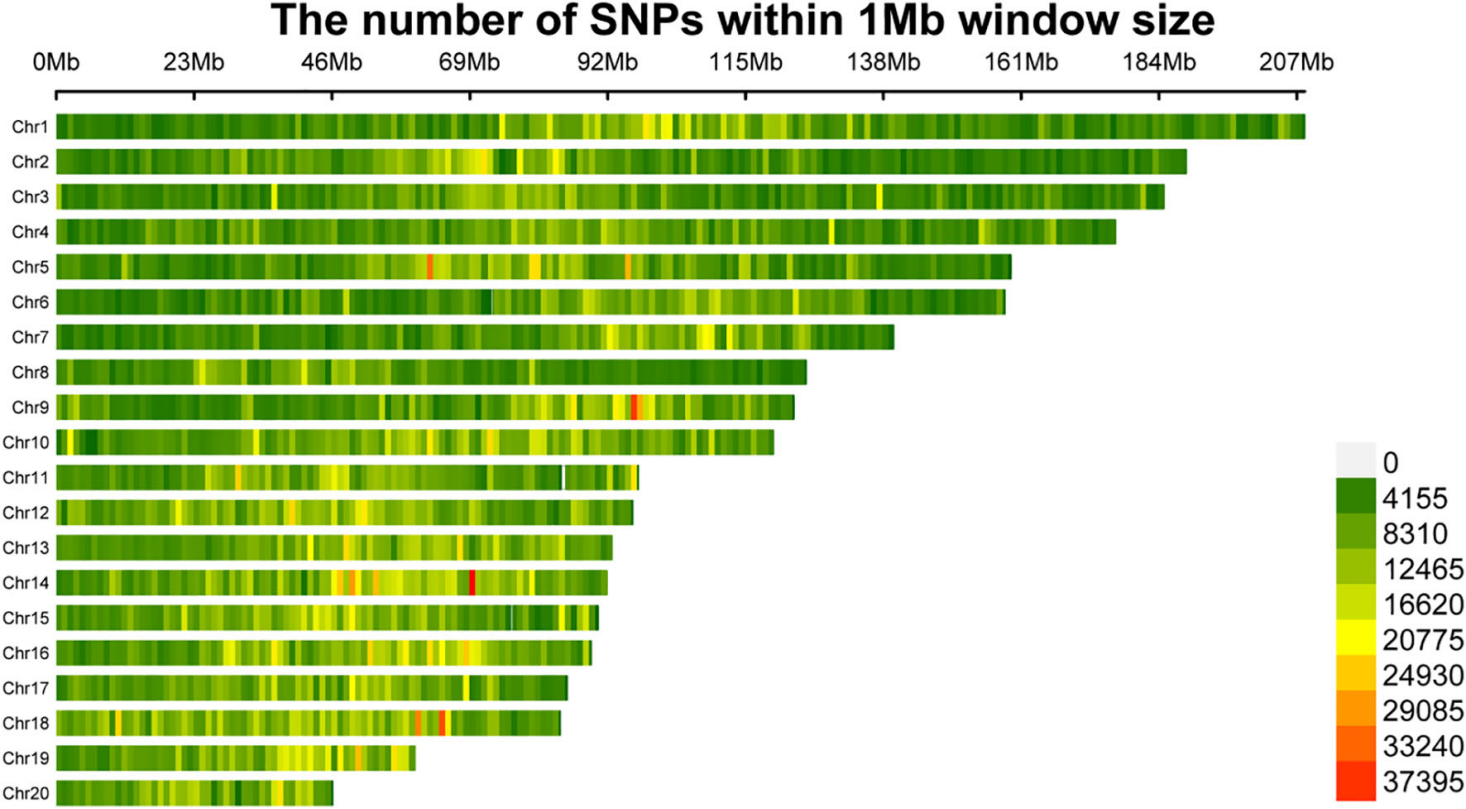
The distribution of SNPs on each chromosomes.

A total of 150 F1 orchid samples were subjected to whole-genome resequencing with an average sequencing depth of 5×, yielding approximately 12 Gb of data per sample. The GC content of the resequencing data exceeded 34% in all samples. Quality control metrics indicated a mean Q30 score of 96% and a mean Q20 score of 98%, reflecting high data quality and reliability after removal of duplicates and contaminants, which meets the requirements for subsequent SNP marker development. After quality filtering, a total of 19,116,970 SNP loci were identified across the 150 samples and genotype imputation was performed. Following linkage disequilibrium-based redundancy pruning, 2,306,434 high-quality SNPs were retained for downstream analyses. Chromosomal distribution analysis of these SNPs revealed that chromosome 1 harbored the highest number of SNPs among all autosomes, whereas chromosome 20 contained the fewest SNPs (**Figure 2**).

### 3.2 Genetic map construction

**Figure 3:**
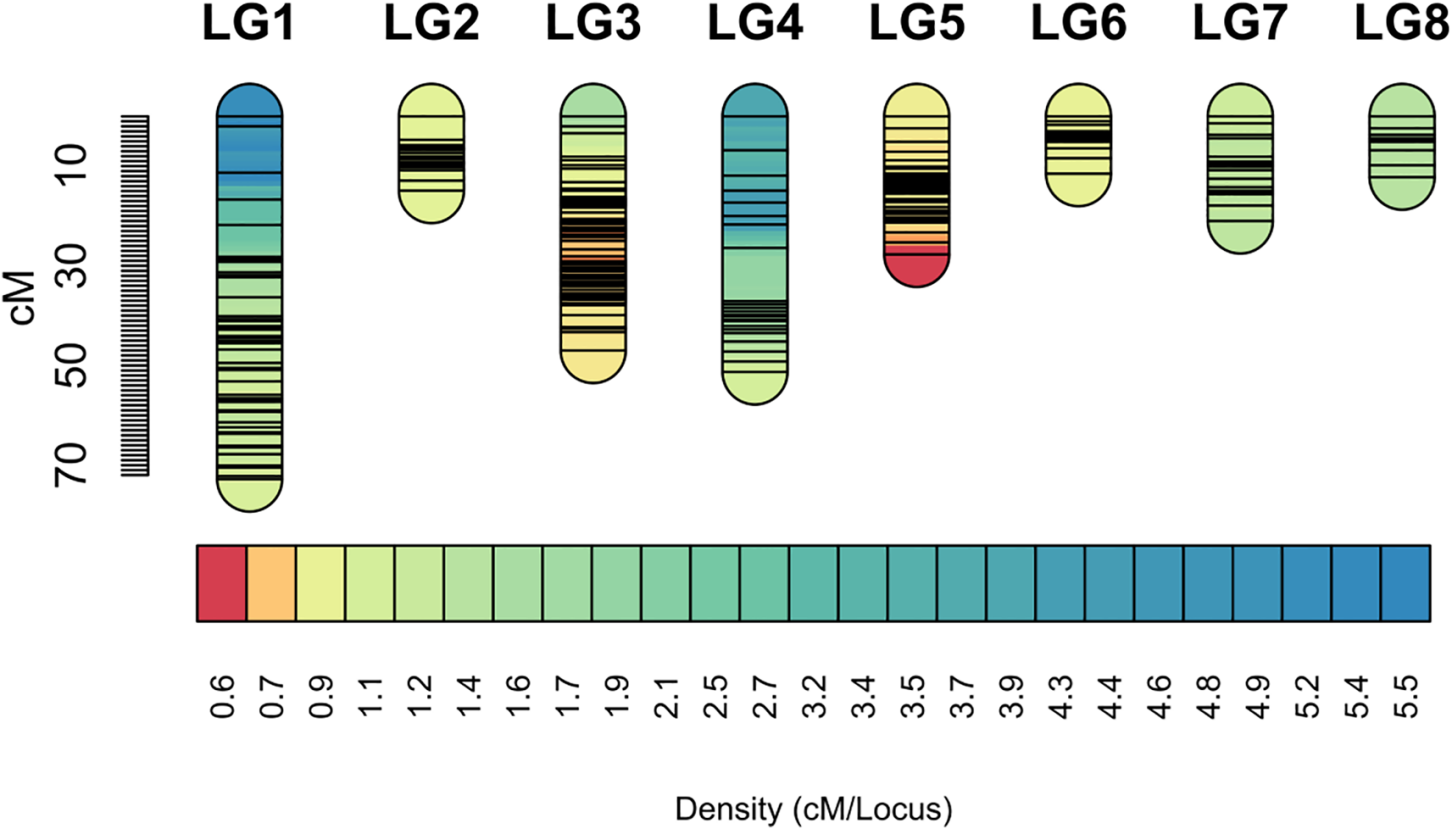
High-density genetic map.

A total of 158,082 SNPs suitable for mapping were initially obtained. These SNPs underwent correction and imputation, resulting in their classification into 7,734 bins. The genetic map was constructed based on these bins. The distribution of SNPs and bins across chromosomes was summarized accordingly. Using the reference genome, bins were assigned to 8 linkage groups. Within each linkage group, marker linear order and genetic distances between adjacent markers were determined using Lep-MAP software. In total, 7,734 markers were developed, culminating in a genetic map with a total length of 255.945 cM. The linkage group (LG) lengths ranged from 11.492 cM to 72.845 cM (**Table 1**), with an average inter-marker distance of 1.19 cM. The longest linkage group was LG1, comprising 45 markers, spanning 72.845 cM with an average marker interval of 1.61 cM. Conversely, the shortest linkage group was LG6, containing 13 markers over 11.492 cM, with an average marker spacing of 0.884 cM (**Figure 3**).

### 3.3 Genome-wide association study of flower color traits based on SNPs

**Figure 4:**
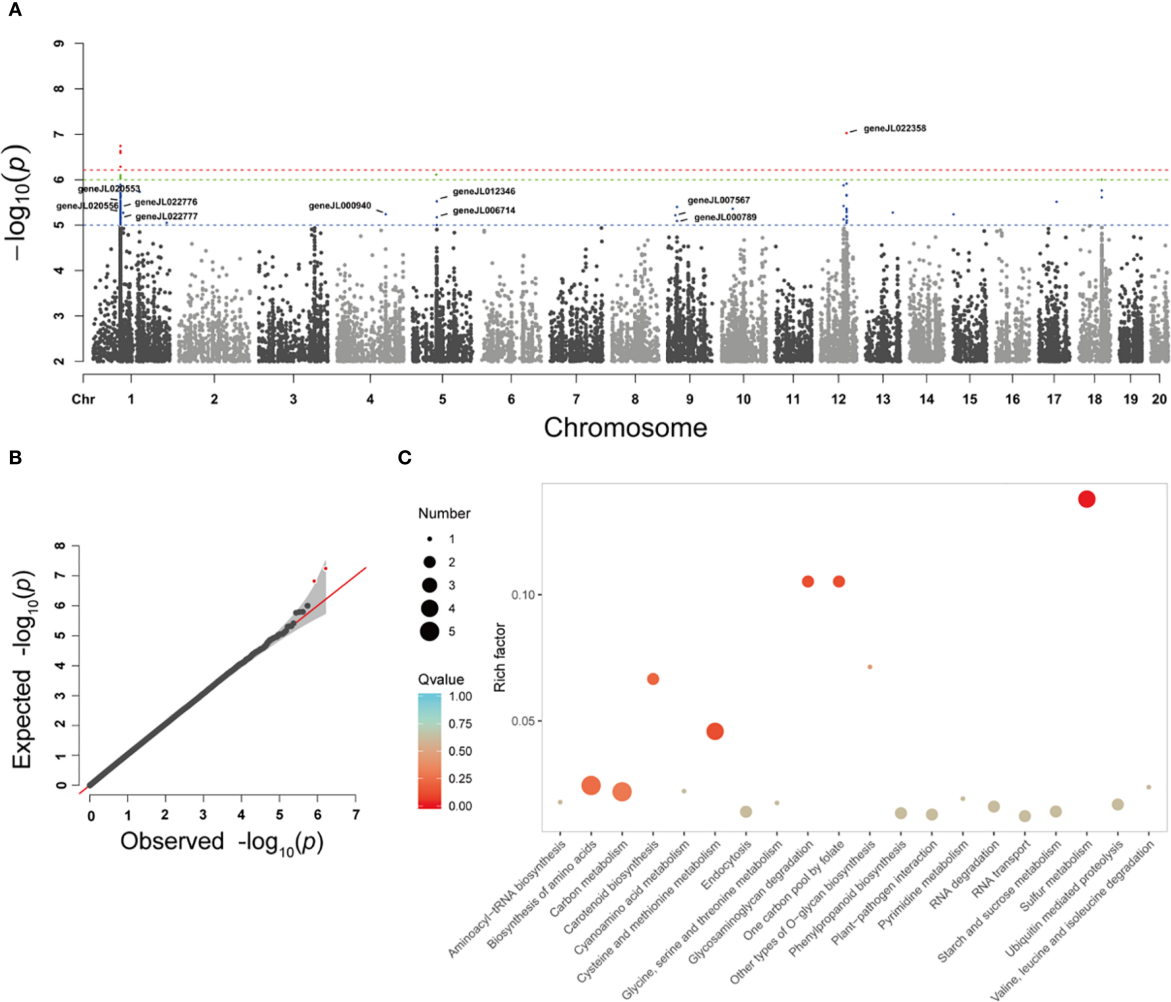
Genome-wide association analysis of orchid flower color. Manhattan map **(A)** and QQ map **(B)** for genome-wide association analysis of orchid flower color traits based on SNP, **(C)** KEGG enrichment analysis of SNPs associated with orchid flower color.

After genotype data quality control, a total of 2,306,434 SNPs were retained for genome-wide association analysis of flower color-related traits in orchids. The GWAS identified 121 SNP loci significantly associated with flower color (*P* < 1×10^-5^), primarily distributed across five chromosomes: chromosomes 1, 4, 5, 9, and 12 (**Figure 4A, B**). Genes located within 0.5 Mb upstream and downstream of these significant SNPs were annotated and subjected to KEGG pathway enrichment analysis. The results revealed significant involvement of these genes in pathways including Aminoacyl-tRNA biosynthesis, Biosynthesis of amino acids, Carbon metabolism, Carotenoid biosynthesis, Cyanoamino acid metabolism, Cysteine and methionine metabolism, and Phenylpropanoid biosynthesis (**Figure 4C**). Gene annotation further identified that geneJL022776 (9-cis-epoxycarotenoid dioxygenase), geneJL006714 (Transcription factor MYB60), geneJL007567 (Carotene epsilon-monooxygenase), and geneJL022709 (WRKY transcription factor 6) are strongly associated with carotenoid biosynthesis and the regulatory mechanisms underlying flower color formation (**Table 2**).

**Table 2:** SNP information table related to suit color.

GeneID	Genename	CHR	Qtl_start	Qtl_end	Description
geneJL022776	geneJL022776	1	74704036	74706008	9-cis-epoxycarotenoid dioxygenase, chloroplastic;
geneJL022777	geneJL022777	1	74678716	74695510	Phototropin-1A;
geneJL020553	geneJL020553	1	74599409	74599615	Protein FAR1-RELATED SEQUENCE 12;
geneJL020556	geneJL020556	1	74609358	74609771	Homeobox protein BEL1 homolog;
geneJL006714	geneJL006714	5	62386891	62387891	Transcription factor MYB60
geneJL007567	geneJL007567	9	20267786	20268769	Carotene epsilon-monooxygenase, chloroplastic;
geneJL000940	geneJL000940	4	128881983	128882228	Transcription factor bHLH62;
geneJL000789	geneJL000789	9	24671567	24700140	WRKY transcription factor 6
geneJL022358	geneJL022358	12	69804163	69824800	F-box/kelch-repeat protein At1g74510;
geneJL012346	geneJL012346	5	63795742	63807554	Ubiquitin carboxyl-terminal hydrolase 9;

### 3.4 RNA-seq global analysis and identification of differentially expressed genes

**Figure 5:**
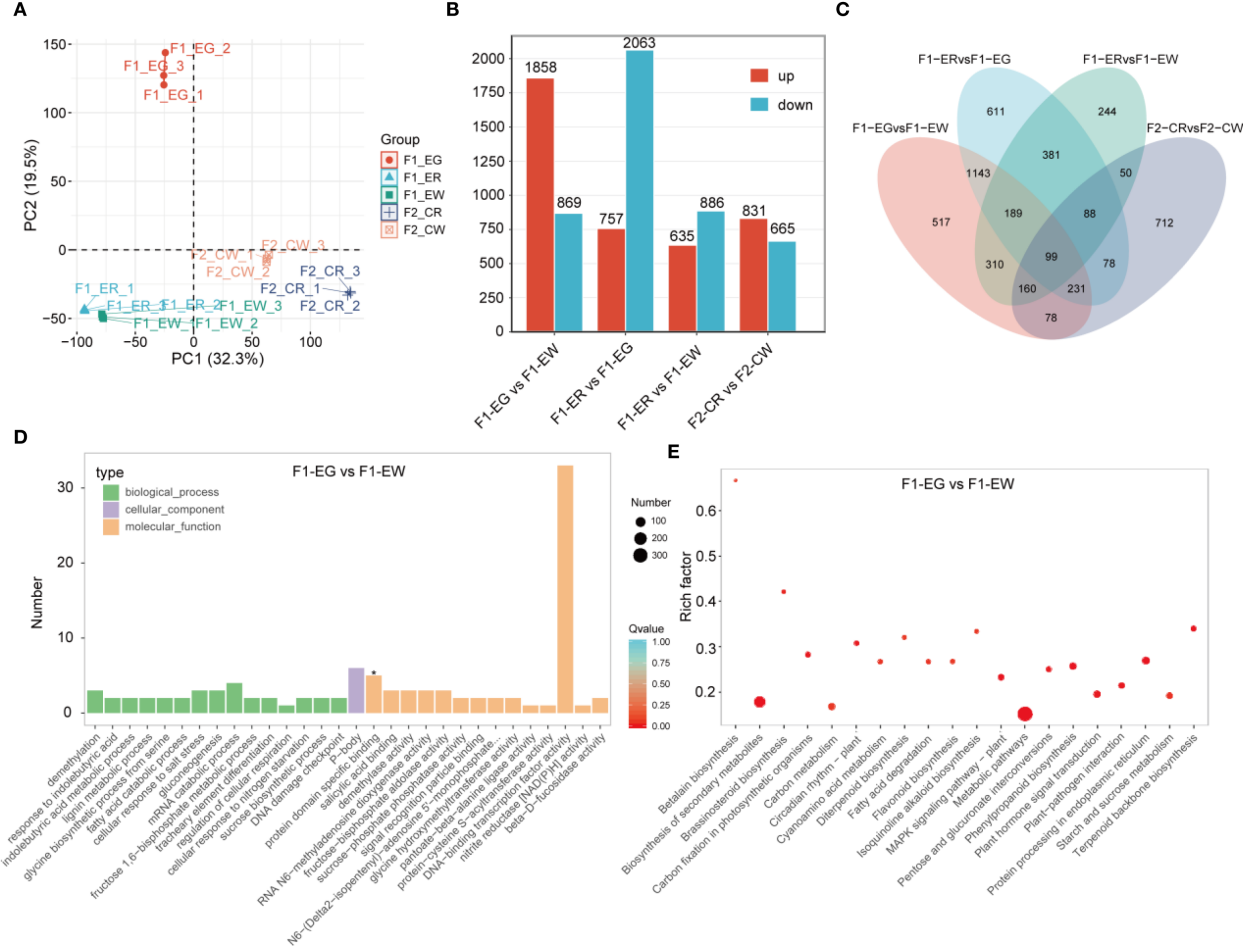
RNA-seqanalysis. Principal component analysis **(A)**, statistics of differentially expressed genes for different comparison groups **(B)**, Venn diagram showing overlaps among the four comparison groups **(C)**, and GO **(D)** and KEGG **(E)** enrichment analyses for the F1-EG vs. F1-EW comparison group.

To elucidate the key regulatory genes controlling flower color in orchids, we performed RNA sequencing (RNA-seq) on petal samples from five different flower color phenotypes. A total of 15 RNA-seq samples generated 177.48 Gb of raw data, with each sample yielding more than 8.37 Gb of clean data. The percentage of bases with Q30 quality scores exceeded 90.41% for all samples, and the mapping rate to the reference genome was higher than 87.20%. Principal component analysis (PCA) demonstrated that biological replicates clustered tightly together, indicating the reliability and reproducibility of the transcriptomic data (**Figure 5A**). To characterize the transcriptomic dynamics under different flower color conditions, differential expression analysis was conducted. In the comparison group F1-EG vs F1-EW, a total of 2,727 DEGs were identified, including 1,858 upregulated and 869 downregulated genes. In the F1-ER vs F1-EG comparison, 2,820 DEGs were detected, comprising 757 upregulated and 2,063 downregulated genes. The F1-ER vs F1-EW group revealed 1,521 DEGs, with 635 upregulated and 886 downregulated genes. In the F2-CR vs F2-CW comparison, 1,496 DEGs were identified, including 831 upregulated and 665 downregulated genes (**Figure 5B**). Venn diagram analysis of all DEGs from the four comparison groups revealed 99 DEGs that were differentially expressed across all flower color phenotypes (**Figure 5C**).

**Figure 6:**
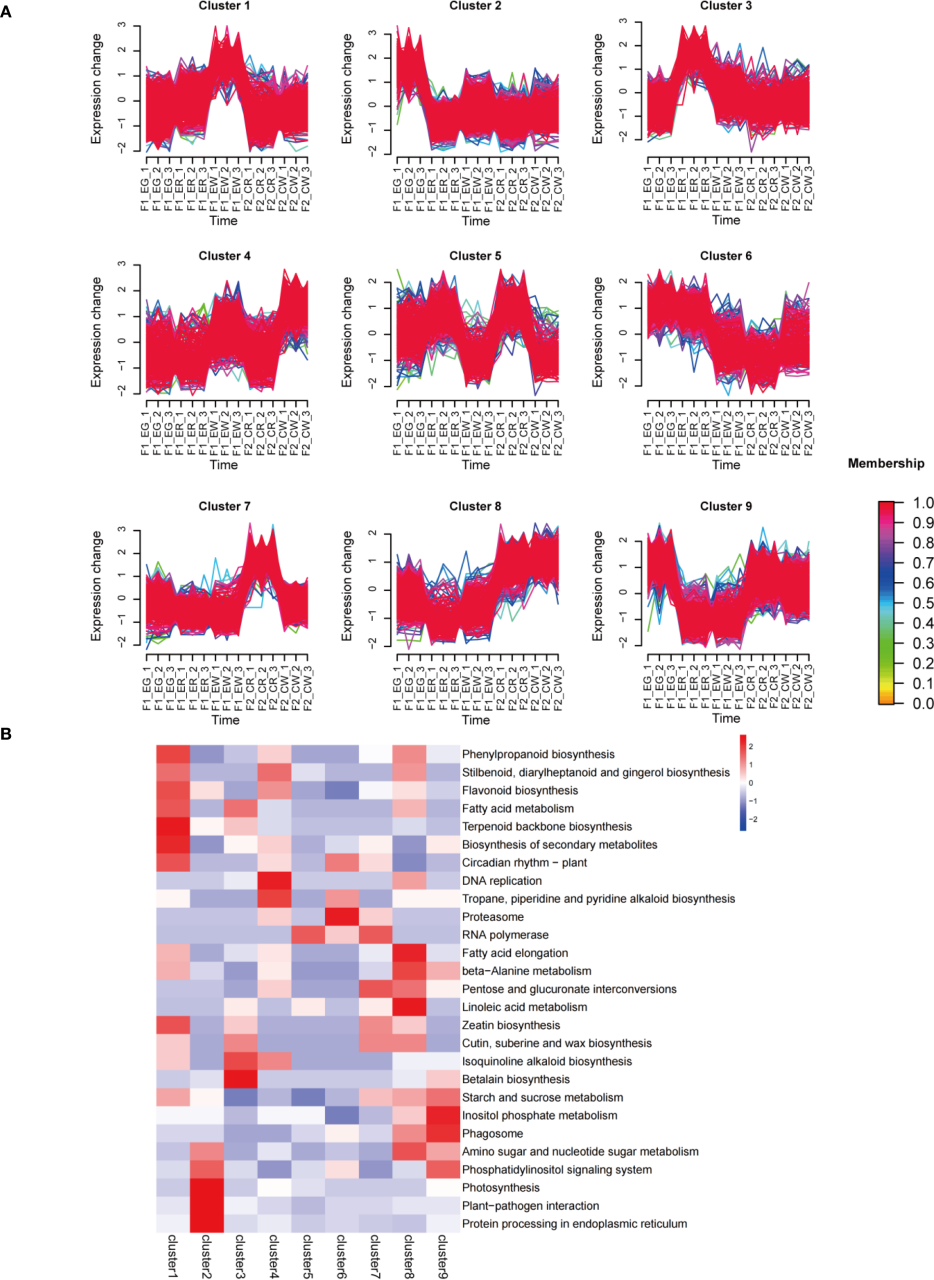
DEG clustering analysis. Nine statistically significant clusters **(A)**, KEGG analysis of genes from the nine clusters **(B)**.

To elucidate the specific functional roles of DEGs in each comparison group, we performed GO and KEGG pathway enrichment analyses on the differential genes from each group (**Figures 5D, E**). GO classification further characterized the functional distribution of DEGs. The molecular function category was predominant, with DNA binding activity representing the largest gene set, highlighting the potential involvement of transcriptional regulation mechanisms. Biological processes such as response to demethylation, indolebutyric acid metabolism, and DNA damage checkpoint were also represented, albeit with fewer genes. Cellular component enrichment was less pronounced, with a limited number of genes associated with specific cellular structures. Notably, protein dimerization activity was marked as statistically significant, implying its possible regulatory role. KEGG pathway enrichment revealed significant enrichment in multiple metabolic pathways, notably betalain biosynthesis, secondary metabolite biosynthesis, brassinosteroid biosynthesis, and carbon fixation in photosynthetic organisms. Among these, the betalain biosynthesis pathway exhibited the highest enrichment factor, indicating a pivotal role in the biological context studied. Additionally, pathways related to circadian rhythm, flavonoid biosynthesis, and MAPK signaling were also significantly enriched, suggesting a complex regulatory network underlying the phenotypic variation. K-means clustering analysis was conducted on 4,891 differentially expressed genes (DEGs), resulting in the identification of nine statistically significant clusters (**Figure 6A**). Each cluster was annotated according to the pathways in which its member genes participate. KEGG pathway enrichment analysis of each cluster revealed that the same significantly enriched pathway may exhibit two distinct expression patterns (**Figure 6B**).

### 3.5 qPCR validation of five key genes

**Figure 7:**
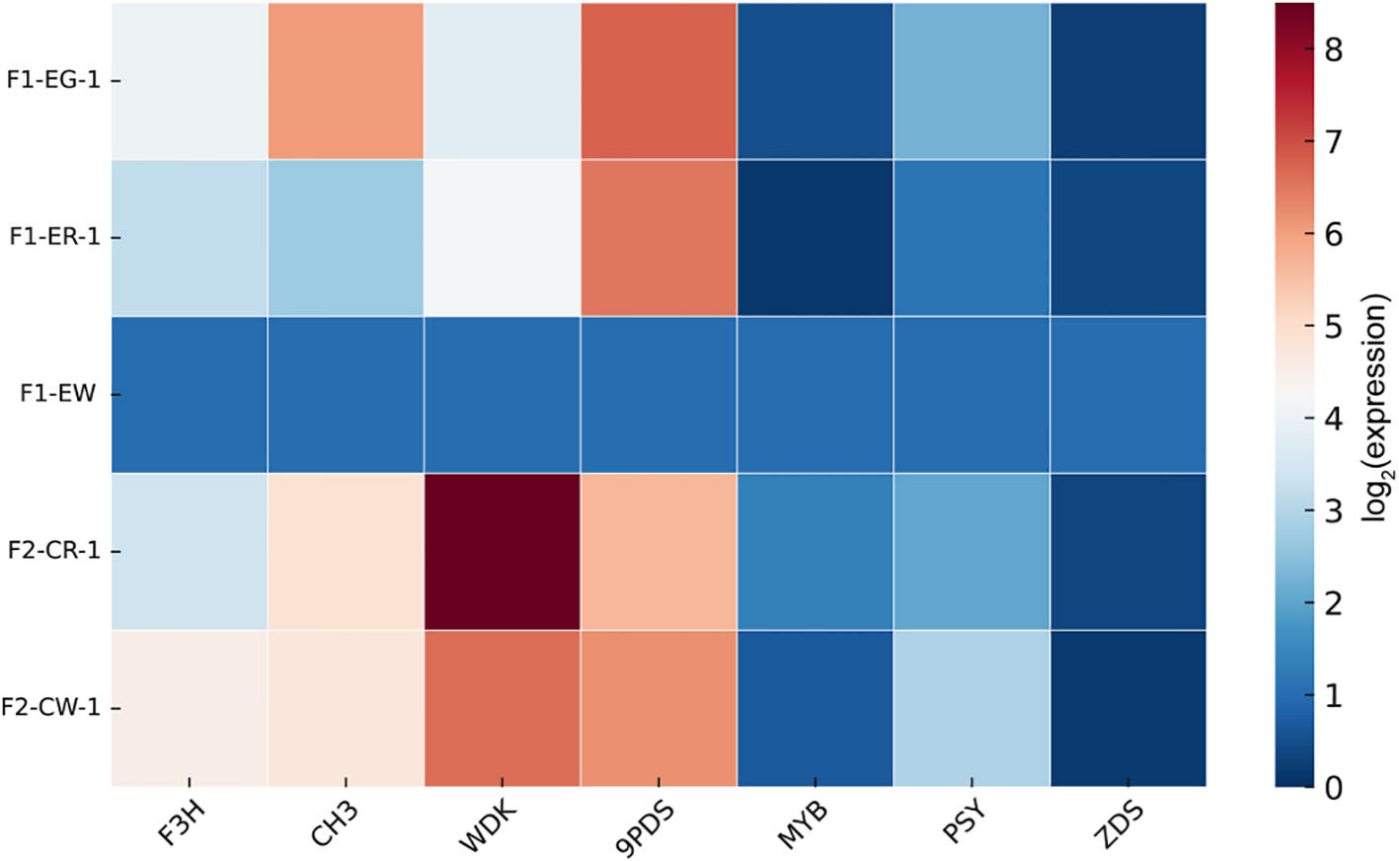
qPCR validation of key genes.

To clarify the roles of carotenoid metabolism and phenylalanine biosynthesis in flower color regulation, we performed qPCR validation on seven key genes involved in these two pathways to assess their expression patterns across flower color tissues (**Figure 7**). The results showed that all seven genes were expressed in the various flower color tissues but exhibited tissue-specific expression and differential expression levels among genes. Notably, genes such as WDK, MYB60, ZDS, and PSY displayed increased expression in white flower samples, whereas F3H, CH3, and PDS showed an opposite expression trend.

## 4 Discussion

In this study, we applied whole-genome resequencing (WGRS) to construct the highest-resolution genetic linkage map reported for orchids, using 150 F1 individuals derived from a cross between *C.eburneum× C. insigne*. Compared with earlier orchid maps based on SSR or RAD-seq markers (Chao et al., 2018), the WGRS-based approach provided a denser and more comprehensive framework. Importantly, the eight linkage groups correspond to the basic chromosome number of orchids, supporting both the reliability and genomic coverage of the map. Rather than focusing on metrics such as map length or marker density, the significance of this strategy lies in its ability to drive downstream analyses. The observed uneven distribution of SNPs, particularly the enrichment on chromosome 1, is consistent with findings in rice (Xue et al., 2008) and rose (Raymond et al., 2018), suggesting possible biological mechanisms such as structural variation or heterogeneity in recombination rates, as well as technical factors such as sequencing read distribution. These results illustrate how high-density genetic maps can simultaneously serve as tools for gene localization and as resources for exploring genome organization.

Using this genetic framework, GWAS identified 121 SNPs (P < 1 × 10⁻^5^) associated with flower color, distributed across five chromosomes. This pattern indicates a polygenic basis for pigmentation in orchids. KEGG enrichment analysis demonstrated that candidate genes were concentrated in carotenoid and phenylpropanoid biosynthesis pathways, aligning with classical models of flower color formation (Yang et al., 2024; Vogt, 2010). The phenylpropanoid pathway, one of the central routes of secondary metabolism, initiates from phenylalanine and proceeds through PAL, C4H, and 4CL to produce coumaroyl-CoA, a precursor for flavonoids and anthocyanins. Anthocyanins depend directly on intermediates of this pathway, but their accumulation is further shaped by competition in metabolic fluxes and by transcriptional control. For example, flavonol synthase (FLS) diverts dihydroflavonols away from anthocyanin biosynthesis, reducing pigment precursors (Luo et al., 2016). Consistent with this, chrysanthemum studies show that FLS overexpression decreases anthocyanin content, while its inhibition enhances pigmentation (Zhou et al., 2021). Similarly, knockout of 4CL3 in rice reduces anthocyanin accumulation in the hull, emphasizing the necessity of this enzyme for flavonoid synthesis (Sun et al., 2013).

Beyond pathway-level enrichment, we identified four candidate genes—NCED, MYB60, LUT1, and WRKY6—that may regulate flower color variation in orchids. NCED, a rate-limiting enzyme in ABA biosynthesis, catalyzes the oxidative cleavage of 9-cis-epoxycarotenoids to yield xanthoxin (Nambara and Marion-Poll, 2005). In addition to its role in ABA metabolism, NCED has been implicated in carotenoid-derived pigmentation, as demonstrated in snapdragon, where NCED activity correlates with yellow pigment accumulation (Ohmiya et al., 2006). Similar patterns were observed in chrysanthemum, where NCED expression is positively associated with carotenoid content (Ohmiya et al., 2006). Moreover, ABA itself may influence anthocyanin stability by modifying vacuolar pH or cellular redox status (Frey et al., 2012).

MYB60, belonging to the R2R3-MYB family, is best known for its function in stomatal regulation and drought responses in Arabidopsis (Cominelli et al., 2005). However, MYB proteins in orchids are functionally diverse. MYB60 may act by binding to the promoters of carotenoid biosynthetic genes such as LUT1 or PSY, thereby promoting yellow pigment accumulation. It may also interact with bHLH or WD40 proteins to regulate anthocyanin biosynthetic genes such as CHS or DFR. Evidence from Phalaenopsis demonstrates that MYB1 activates ANS and DFR expression, driving purple pigmentation (Fu et al., 2019). In Arabidopsis, MYB75 (PAP1), a homolog of MYB60, directly activates anthocyanin biosynthesis genes, enhancing red pigmentation (Gonzalez et al., 2008).

LUT1 encodes carotene ϵ-monooxygenase, which catalyzes ϵ-ring hydroxylation of carotenoids, converting lycopene to δ-carotene and ultimately lutein (Li et al., 2009). As lutein is a major yellow pigment in petals, LUT1 expression directly impacts coloration. In Oncidium orchids, high LUT1 expression correlates with increased lutein content, with yellow cultivars showing threefold higher expression compared with white flowers (Chiou et al., 2010). In tomato, LUT1 mutants lack lutein in leaves and fruits, leading to pale phenotypes (Galpaz et al., 2006).

WRKY6, a member of the WRKY transcription factor family, is typically associated with stress responses. However, studies suggest it may also affect pigmentation. In grapevine, WRKY26 directly binds the promoters of CHS and DFR, enhancing anthocyanin accumulation (Guillaumie et al., 2011). In petunia, PhWRKY44 represses the anthocyanin transporter PhAN9, reducing vacuolar pigment accumulation and leading to paler flowers. In Arabidopsis, overexpression of WRKY6 upregulates anthocyanin biosynthesis genes, such as DFR, and produces purple-red leaves (Robatzek and Somssich, 2002). These findings suggest that WRKY6 may influence orchid flower color by modulating chromatin accessibility and transcriptional regulation of anthocyanin pathway genes.

Taken together, our findings demonstrate that WGRS-based high-density genetic mapping provides a powerful approach for studying complex traits in orchids. By combining linkage mapping and GWAS, we identified key SNPs, enriched pathways, and candidate regulators associated with flower pigmentation. This not only validates the utility of WGRS for genetic dissection in orchids but also expands our understanding of the molecular basis of floral coloration.

## 5 Conclusion

In this study, the F_1_ population obtained by crossing *C.eburneum× C. insigne* was successfully constructed by whole genome resequencing technology, containing 7,734 bin markers, with a total map distance of 255.945 cM and an average marker interval of only 1.19 cM. Based on this map, we identified 121 SNP sites significantly related to flower color through genome-wide association analysis (GWAS) system, and found that these loci are mainly enriched in carotenoid biosynthesis and phenylpropane metabolism pathways. Further transcriptome analysis and qPCR verification were used to screen out a number of key candidate genes, including NCED, MYB60, LUT1 and WRKY6, which may jointly affect orchid flower color formation by regulating pigment synthesis and accumulation. This study not only realizes high-precision genetic mapping based on whole genome resequencing in orchids for the first time, providing an important tool for the genetic analysis of complex traits such as flower color, but also deeply reveals the molecular basis of orchid flower color formation, providing valuable genetic resources and theoretical basis for future orchid molecular breeding. In addition, the established high-throughput genotyping and phenotypic association analysis strategy can also provide a reference for other genetic studies of non-model plant traits.

## Data Availability

The datasets presented in this study can be found in online repositories. The names of the repository/repositories and accession number(s) can be found here: PRJNA1356820.
